# Preliminary Data on the Senolytic Effects of *Agrimonia pilosa* Ledeb. Extract Containing Agrimols for Immunosenescence in Middle-Aged Humans: A Randomized, Double-Blind, Placebo-Controlled, Parallel-Group Comparison Study

**DOI:** 10.3390/nu17040667

**Published:** 2025-02-13

**Authors:** Yoshiki Shimizu, Shieri Shimodan, Mariko Hayashida, Misato Yazaki, Tsuyoshi Sakurada, Tomomichi Watanabe, Yuri Ishii, Yoshie Hirose, Jiro Saito, Sachiyuki Teramoto

**Affiliations:** 1 FANCL Research Institute, FANCL Corporation, 12-13 Kamishinano, Totsuka-ku, Yokohama 244-0806, Japan; shieri2104@fancl.co.jp (S.S.); mariko1512@fancl.co.jp (M.H.); misato_110401@fancl.co.jp (M.Y.); sakurada_tsuyoshi@fancl.co.jp (T.S.); watanabe_tomomichi@fancl.co.jp (T.W.); yuishii@fancl.co.jp (Y.I.); sateramoto@fancl.co.jp (S.T.); 2 Yukeikai Medical Corporation Ginza Yoshie Clinic, V88 Building 5F, 2-5-11 Ginza, Chuo-ku, Tokyo 104-0061, Japan; yh@ginzabiyou.com; 3 Medical Station Clinic, 3F Ichikawa Gakugei-dai Building, 3-12-8 Takaban, Meguro-ku, Tokyo 152-0004, Japan; j.saito@med-station.jp

**Keywords:** *Agrimonia pilosa* Ledeb. extract, agrimol, immunosenescence, senolytic agent, senescence-associated β-galactosidase

## Abstract

Objectives: To assess the effects of agrimol-containing *Agrimonia pilosa* Ledeb. extract (APE) for senescent immune cell removal in middle-aged Japanese adults with immunosenescence. Design and Setting: A randomized, double-blind, placebo-controlled, parallel-group study was conducted in Japan between June 2023 and April 2024. Participants: 110 individuals aged 40–59, selected based on CD8+ T cells with highly-expressing-senescence-associated-β-galactosidase (SA-βGal). Intervention: Participants were randomly assigned to receive 50 mg APE containing 0.2 mg of agrimols or a placebo for eight consecutive weeks. Measurements: The primary endpoint was the change in the proportion of CD8+ T cells with high SA-βGal expression at 8 weeks of intake from the baseline. The secondary endpoints included the proportion of CD4+ T cells with high SA-βGal expression, CD4+ and CD8+ T cell subsets, and the ratio of various immune cells. Results: Of the 635 subjects screened, 110 with immunosenescence were included in this study. In total, 55 participants in the placebo group and 53 in the APE group completed the intervention. There were no statistically significant changes in either the primary or secondary endpoints due to APE intake. In the male population, the proportion of CD8+ T cells with high SA-βGal expression was reduced by APE intake (*p* = 0.044). Furthermore, the proportion of naïve CD8+ T cells increased and the number of effector memory CD8+ T cells decreased with the consumption of APE. Conclusions: APE was suggested to reduce senescent immune cells, indicating its potential as a candidate senolytic agent for humans; however, the results of this study are preliminary data, and further research on APE is needed (clinical trial registration: UMIN000051574).

## 1. Introduction

In recent years, the global population has been growing older, and it is estimated that the proportion of the world’s elderly aged 65 years or older will reach 16% by 2050, compared to approximately 10% in 2022 [1]. While the average lifespan is increasing, the number of elderly people with physical, mental, and social problems due to the aging process is also increasing, as is the risk of death and the need for nursing care for the elderly [2,3,4]. Aging, or senescence, is a process that typically begins around the age of 40 and is driven by genetic and environmental factors that cause a gradual decline in physiological functions [5,6,7].

Senescence is defined as an irreversible pathophysiological process involving the progressive decline of mental and physical functions in postmature organisms [5,6]. Cellular senescence, a key hallmark of aging, is a state in which cells cease to proliferate irreversibly due to various stressors and plays an important role in suppressing tumorigenesis, wound healing, and tissue repair [8]. Although senescent cells provide these beneficial functions, their accumulation leads to tissue dysfunction and chronic inflammation through the senescence-associated secretory phenotype (SASP), which contributes to various aging-related diseases [9,10]. Therapeutic strategies targeting senescent cells, such as senolytic agents, have shown promise in animal models. Senolytic agents selectively remove senescent cells, alleviating age-related disorders and extending lifespan by reducing the secretion of SASP factors [7,11,12,13,14]. These findings underscore the importance of further investigating senolytic therapies as a means of mitigating the negative effects of aging.

One of the key manifestations of aging is immunosenescence, which refers to the age-related decline in immune function. Immunosenescence is characterized by thymic atrophy, a decrease in the proportion of naïve T cells, an increase in memory T cells, and heightened chronic inflammation [15]. These changes are hallmarks of immunosenescence and increase the risk of infection and other diseases in elderly individuals [16,17]. Animal studies highlight the harmful effects of immunosenescence, such as accelerated aging and reduced lifespan due to mitochondrial dysfunction in T cells [18]. Perforin-knockout mice with deficient immune surveillance accumulate more senescent cells with age compared to control mice. These mice develop multiple age-related diseases and have a reduced survival rate [19]. In other words, the accumulation of senescent immune cells during immunosenescence may be a risk factor not only for disease but also for aging.

Among senescence markers, senescence-associated β-galactosidase (SA-βGal) is widely used due to its high expression in senescent cells. While not its optimal pH, SA-βGal activity can be detected at pH 6.0 and serves as an indicator of increased lysosomal mass in senescent cells [20,21,22]. Martínez-Zamudio et al. demonstrated the utility of fluorescence-activated cell sorting (FACS) technology in identifying senescent immune cells. Their study showed that older adults have a higher proportion of high-SA-βGal-expressing CD8+ T cells, which are characterized by telomere dysfunction, elevated p16 expression, and p16-induced senescence [23].

In our research group, we utilized FACS to sort peripheral blood CD8+ T cells into high- and low-SA-βGal-expressing cell populations and performed preliminary analysis (unpublished data). The results showed that high-SA-βGal-expressing CD8+ T cells displayed significantly higher p16 expression compared to low-SA-βGal CD8+ T cells, with the observations reported by Martínez-Zamudio et al. [23]. These findings suggest that SA-βGal is a reliable marker of senescence. In other words, measuring SA-βGal expression in peripheral blood CD8+ T cells allows for a minimally invasive evaluation of senolytic therapies for immunosenescence.

*Agrimonia pilosa* Ledeb., also known as Pilosa Cocklebur, is a plant of the family Rosaceae traditionally used in Chinese medicine. It contains many bioactive compounds, such as phloroglucinols, flavonoids, tannins, phenols, triterpenoids, and organic acids [24,25,26]. *A. pilosa* Ledeb. extract (APE) has been reported to have medicinal effects, such as antioxidant, anti-inflammatory, antibacterial, antitumor, and anti-diabetic effects, and is attracting attention as a food material with anti-aging effects [27,28,29,30,31]. APE and agrimol B, a phloroglucinol derived from *A. pilosa* Ledeb., have been shown to induce apoptosis in doxorubicin-induced senescent cells and reduce senescence markers such as p16 and SASP factors in aged mice [32]. Watanabe et al. reported that APE administration in aged mice led to a reduction in high-SA-βGal CD8+ T cells and a decrease in memory CD8+ T cells and promoted an increase in naïve CD8+ T cells, suggesting its potential to alleviate immunosenescence [32]. These findings indicate that APE may mitigate immunosenescence, making it a promising candidate as a senolytic agent.

However, the senolytic effects of APE in humans remain unclear. Therefore, we conducted a randomized, double-blind, placebo-controlled parallel-group comparison study to obtain preliminary data on the senolytic effects of APE-containing agrimols on senescent immune cells in middle-aged Japanese men and women with immunosenescence using FACS technology with SA-βGal as a marker. We also investigated potential sex differences in the effects of APE.

## 2. Materials and Methods

### 2.1. Study Setting

This study was reviewed and approved (approval date: 22 June 2023; review number: 230622-1; principal investigators: Sayuri Matsuoka [until 30 September 2023] and Sachiyuki Teramoto [from 1 October 2023]) by the Medical Station Clinic Ethics Review Committee, which is composed of unrelated third parties (IRB number: 20000022). This study was conducted following the Declaration of Helsinki (revised in October 2013) and in accordance with the Ethical Guidelines for Medical and Health Research Involving Human Subjects (Notification No. 1 by the Ministry of Education, Culture, Sports, Science and Technology; Ministry of Health, Labour and Welfare; and Ministry of Economy, Trade and Industry; 23 March 2021 [partially revised on 27 March 2023]) and the Guidance for Ethical Guidelines for Medical and Health Research Involving Human Subjects (Ministry of Education, Culture, Sports, Science and Technology; Ministry of Health, Labour and Welfare; and Ministry of Economy, Trade and Industry; 16 April 2021 [partially revised on 17 April 2023]). Before this study, the principal investigational doctor thoroughly explained the purpose and details of the study to the participants. Written informed consent was obtained from all participants, and only those who provided consent were included.

This study was conducted using the participant management and research implementation systems provided by the Medical Station Clinic and Yukeikai Medical Corporation Ginza Yoshie Clinic. The principal investigational doctor (Jiro Saito) and one of the investigational doctors (Yoshie Hirose) supervised all research-related activities, including providing instructions to the participants of this study, obtaining consent, conducting interviews, and managing the examination system. The principal investigational doctor confirmed and assessed the adverse events, prepared case reports, and administered the necessary treatments for adverse events, as needed. This study was conducted between June 2023 and April 2024. Prior to the commencement of the trial, the study details were registered with the University Hospital Medical Information Network Clinical Trials Registry (UMIN-CTR) (clinical trial registration: UMIN000051574).

### 2.2. Subjects

Subject characteristics such as medical history, drinking and eating habits, clinical and physical examinations, and peripheral blood mononuclear cell (PBMC) tests were conducted to screen 635 participants, whose consent was obtained in writing prior to study participation. Based on the screening test results, 110 participants who met the selection criteria and did not meet the exclusion criteria were selected. Participants were enrolled in order of the proportion of their high-SA-βGal-expressing CD8+ T cells (high-SA-βGal CD8+ T cells) from among the group of prospective participants.

Participants were selected based on the following criteria: Japanese male and female participants aged 40–59 years at the time of obtaining consent, participants with a relatively high proportion of high-SA-βGal CD8+ T cells, subjects who could provide consent, and those who did not meet the exclusion criteria. The exclusion criteria were participants who had undergone surgical resection of the gastrointestinal tract (not including appendectomy); participants who had a disease that required constant medication or those with a serious medical history that required medication; participants with autoimmune diseases or a history of autoimmune diseases; participants with mental illness, chronic fatigue syndrome, insomnia, or a history of such illnesses; patients diagnosed with or suspected of having sleep apnea; participants undergoing treatment related to sleep, stress, and/or fatigue; night-and-day-shift workers or manual laborers; participants who regularly took or planned to take medicines, supplements, Foods for Specified Health Uses (FOSHU), and/or foods that may have affected the results during the study; participants with possibilities of emerging allergies related to the study intervention; heavy drinkers and excessive smokers; participants who were planning to travel abroad during the study period or who were planning a long-term domestic trip for more than one week in the study period; participants who were judged unsuitable for the study based on the results of the lifestyle questionnaire; participants who were judged unsuitable for the study based on the results of their clinical laboratory or cardiopulmonary function tests; participants whose physical measurements, physical examination values, and clinical examination values before the start of intake were significantly outside the reference range; participants who were participating in other clinical studies at the start of the study; participants who were pregnant or intended to become pregnant; and participants whom the investigator judged as unsuitable for the study for other reasons.

### 2.3. Sample Size Calculation

Based on data from a previous study using APE (UMIN000048415), we calculated that a sample size of 55 participants per group (totaling 110 subjects) would be necessary for this study. This calculation accounted for an anticipated difference of 4.6% in the change in the proportion of high-SA-βGal CD8+ T cells between the APE group and the placebo group, with a common standard deviation of 8.1%. This study assumed a two-sided significance level of 5% and a power of 0.8 for the Student’s *t*-test, considering potential dropout cases during the trial or exclusions due to protocol violations.

### 2.4. Trial Intervention

The APE used in this study was prepared by Maruzen Pharmaceuticals Co., Ltd. (Hiroshima, Japan) from dried *A. pilosa* Ledeb. The extraction process involved using aqueous ethanol to extract the active components, removing water-soluble substances, concentrating hydrophobic components containing agrimols, and then drying and pulverizing them with excipients. The specification for the APE ensured that it contained at least 0.4% agrimols.

The test food consisted of hard capsules containing 50 mg APE per capsule. Each 50 mg of APE contained 0.2 mg of agrimols, including agrimol B. The placebo capsules were identical in appearance to the test food capsules. They contained cellulose instead of APE and were colored similarly to ensure indistinguishability from the test food. The test food was administered to the subjects in the form of one capsule (50 mg APE or placebo) per day, taken with water or warm water.

In studies evaluating the effects of immunosenescence, interventions are sometimes conducted over a period of approximately four to eight weeks [33,34,35,36]. Additionally, from our research group’s previous study involving APE intake (UMIN000048415), it was considered that the intervention effects of APE could be observed within about eight weeks. Therefore, the intake period for the study food in this research was set to eight weeks (56 days).

### 2.5. Trial Protocol

This was a randomized, double-blind, placebo-controlled, parallel-group comparison study. The randomization of participants into the APE and placebo groups was performed by Yoshihisa Kibune (Consultant, EP Mediate Co., Ltd., Tokyo, Japan) using computer-generated random numbers based on the participants selected during screening. Blinding was applied to all parties involved in this study (participants, intervention administrators, and evaluators), and the code was not broken until the analysis population was fixed.

After randomization, the subjects were brought in for baseline tests, which included weight measurement, blood pressure and pulse measurement, blood sampling (for peripheral blood mononuclear cells [PBMCs] and serum), urine collection, medical interviews, and questionnaire surveys. Subjects who completed the baseline examinations were administered APE or the placebo and began their intake. Starting from the first day of intake, participants returned for follow-up visits at 4 and 8 weeks, where they underwent weight measurement, blood pressure and pulse measurement, blood sampling (for PBMC and serum), urine collection, medical interviews, and questionnaire surveys. The specified examination days for the 4-week and 8-week assessments were set as days 29 and 57, respectively, and the baseline examination was counted as day 1. However, the principal investigational doctor could reschedule the examinations within seven days before or after the specified examination days if necessary. During the intake period, participants were asked to record their intake of trial supplements, exercise, alcohol consumption, physical condition, and use of medications.

The investigational doctors instructed the subjects not to change their lifestyle habits from the time of consent until the end of the intervention period. If any changes in the subjects’ lifestyle habits were observed in their records, the investigational doctors provided guidance to ensure that the subjects maintained their original lifestyle habits.

Blood tests included counts and measurements of white blood cells, red blood cells, hemoglobin, hematocrit, MCV, MCH, MCHC, platelets, total protein (TP), albumin (Alb), A/G ratio, total bilirubin (TB), direct bilirubin (D-B), indirect bilirubin (I-B), ALP at screening and at 8 weeks of intake, AST (GOT), ALT (GPT), LD, γGT (γGTP), total cholesterol (TC), triglycerides (TG), HDL cholesterol (HDL-C), LDL cholesterol (LDL-C), urea nitrogen (UN), creatinine (Cr), uric acid (UA), Na, K, Cl, blood sugar (GLU), HbA1c, insulin, Fe, total iron binding capacity (TIBC), unsaturated iron binding capacity (UIBC), and cystatin C. Urinalysis was performed for protein (qualitative), sugar (qualitative), and occult blood reaction (qualitative). Blood tests and urinalyses were performed by LSI Medience Corporation (Tokyo, Japan) using a standardized method.

### 2.6. Peripheral Blood Mononuclear Cell (PBMC) Collection and Preservation

To collect PBMCs, blood was drawn from participants using BD Vacutainer^®^ CPT™ Mononuclear Cell Preparation Tubes with Sodium Citrate (BD Biosciences, Franklin Lakes, NJ, USA). Blood was drawn for PBMC collection in 8 mL volumes on each test day. After blood collection, the samples were centrifuged to isolate PBMCs. The PBMC layer was then collected into 15 mL tubes, resuspended in PBS(-) (Thermo Fisher Scientific, Inc., Waltham, MA, USA), and centrifuged again. After removing the supernatant, the cells were resuspended in Bambanker^®^ medium (NIPPON Genetics Co., Ltd., Tokyo, Japan) and stored at −80 °C until use.

### 2.7. Flow Cytometric Analyses

Cell staining was performed as previously described, with some modifications [23]. Bafilomycin A1 and SPiDER-βGal included in the Cellular Senescence Detection Kit-SPiDER-βGal (Dojindo Laboratories, Kumamoto, Japan) were dissolved in 30 μL and 20 μL of dimethyl sulfoxide (FUJIFILM Wako Pure Chemical Corporation, Osaka, Japan), respectively. After thawing cryopreserved 5 × 10^5^ human PBMCs and washing them with FACS Buffer (PBS(-), 2% FBS (*v*/*v*) (Thermo Fisher Scientific, Inc.), 2 mM EDTA (Thermo Fisher Scientific, Inc.) and 1% penicillin-streptomycin (Sigma Aldrich, St. Louis, MO, USA)), the PBMCs were treated with bafilomycin A1 diluted 1:500 in HBSS(-) (FUJIFILM Wako Pure Chemical Corporation) at 37 °C for 1.5 h. In addition to SA-βGal, endogenous βGal was also present in the cells and was expressed regardless of senescence. SPiDER-βGal reacted with both βGals, but with bafilomycin A1 treatment, which inhibited only endogenous βGal, only SA-βGal was specifically detected.

The PBMCs were treated with both SPiDER-βGal and bafilomycin A1 (diluted 1:500 in HBSS(-)) at 37 °C for another 30 min. After washing with FACS Buffer, the PBMCs were treated with human TruStain FcX™ (Biolegend, San Diego, CA, USA, diluted 1:100) on ice for 20 min. After treatment with PE-conjugated anti-human CD3 antibody (SK7, 1:100), Brilliant Violet 510™-conjugated anti-human CD4 antibody (SK3, 1:100), PerCP/Cyanine5.5-conjugated anti-human CD8a antibody (RPA-T8, 1:100), Alexa Fluor^®^ 647-conjugated anti-human CD45RA antibody (HI100, 1:5000), Brilliant Violet 421™-conjugated anti-human CD197 antibody (CCR7) (G043H7, 3:100), and PE/Cyanine7-conjugated anti-human CD279 (PD-1) antibody (EH12.2H7, 1:100) (all from Biolegend, San Diego, CA, USA), the PBMCs were incubated on ice for 30 min. The PBMCs, which were stained for SA-βGal using the same procedure as described above, were also treated with Alexa Fluor^®^647-conjugated Mouse IgG2b, k Isotype Ctrl (MPC-11, 1:50000), Brilliant Violet 421™-conjugated Mouse IgG2a, k Isotype Ctrl (MOPC-173, 21:500), PE/Cyanine7-conjugated Mouse IgG1, and k Isotype Ctrl (MOPC-21, 1:100) (all from Biolegend), used as isotype controls. After washing the antibody-stained PBMCs with FACS Buffer, the PBMCs were treated with Fixable Viability Dye eFluor™ 780 (Thermo Fisher Scientific, Inc., 1:1000) on ice for 20 min. The stained PBMCs were analyzed using a BD FACS Celesta instrument (BD Biosciences) and FlowJo software v10.8.1 (FlowJo, LLC, Ashland, OR, USA). The PBMCs for flow cytometric analysis were acquired at 100,000 events. Each subset of CD4+ and CD8+ T cells was identified using the following markers based on the isotype controls:

- Naïve T cells: CD197(CCR7)+CD45RA+
- Central memory T cells: CD197(CCR7)+CD45RA-
- Effector memory T cells: CD197(CCR7)-CD45RA-
- Terminally differentiated effector memory T cells re-expressing CD45RA (TEMRA): CD197(CCR7)-CD45RA+
- PD-1-positive cells: CD279+

To quantify the percentage of T cells expressing high levels of SA-βGal, we took advantage of the fact that donors often had separate populations consisting of cells with low and high SA-βGal fluorescence. Specifically, we set a gate at the intersection of the two populations; the high population with low signal intensity was designated as “SA-βGal low” and the population with high signal intensity was designated as “SA-βGal high”.

### 2.8. Digital PCR for Gene Expression of Cell Cycle Markers and Sirt1 in PBMCs

Digital PCR (dPCR) was conducted to assess the gene expression levels of cell cycle markers (p16, p21, p53) and Sirtuin 1 (Sirt1).

Total RNA was extracted and purified from 1 × 10^6^ PBMCs using the RNeasy 96 Kit (QIAGEN, Venlo, The Netherlands) according to the manufacturer’s instructions. cDNA was synthesized from purified total RNA by reverse transcription using a PrimeScript™ RT Reagent kit (Takara Bio, Inc., Shiga, Japan).

Absolute quantification of RNA levels was conducted using QIAcuity digital PCR (QIAGEN, Venlo, The Netherlands) with the QIAcuity Nanoplate 8.5 K 96-well (QIAGEN). dPCR was performed with TaqMan probes (Applied Biosystems, Foster City, CA, USA) targeting the following genes: *Actb* (assay ID: Hs01060665_g1), *CDKN2A* (Hs00923894_m1), *CDKN1A* (Hs00355782_m1), *TP53* (Hs01034249_m1), and *Sirt1* (Hs01009006_m1). For each well, 12 μL of reaction solution was prepared, including 3 μL of 4x Probe PCR Master Mix (Takara Bio Inc., Shiga, Japan), 0.4 μM TaqMan Probe, and cDNA template. The thermal cycling program consisted of 20 s at 95 °C, 45 cycles of 1 s at 95 °C, and 20 s at 60 °C. The fraction of target gene for each sample was determined by dividing the number of copies/μL of the target gene by the number of copies/μL of the *Actb* gene. Samples with insufficient template RNA or issues with the accuracy of measurement results were treated as missing values.

### 2.9. Outcome Measures

#### 2.9.1. Efficacy Evaluation

The primary endpoint of this study was the change in the proportion of high-SA-βGal CD8+ T cells in human PBMCs from baseline to week 8.

The secondary endpoints of this study included changes from baseline to week 8 in the following parameters: the proportion of high-SA-βGal CD4+ T cells in human PBMCs; the proportion of naïve cells, central memory cells, effector memory cells, TEMRA cells, and PD-1 expressing cells among both CD8+ and CD4+ T cells in human PBMCs; and the ratios of CD3+ T cells/lymphocytes, CD4+ T cells/lymphocytes, CD8+ T cells/lymphocytes, and CD4+/CD8+ T cells in human PBMCs.

In addition, as a subgroup analysis specified in the study protocol, efficacy evaluations were conducted according to sex.

#### 2.9.2. Exploratory Efficacy Evaluation

As an assessment of senescence markers other than SA-βGal, the expression levels of p16, p21, p53, and Sirt1 in the PBMC population were evaluated.

#### 2.9.3. Safety Evaluation

Safety was assessed by evaluating the test values of each examination item at week 8 and any adverse events that occurred after the intake of the trial supplements.

### 2.10. Statistical Analysis

The APE and placebo groups for each item were evaluated using Student’s *t*-test. Analysis of covariance (ANCOVA) was performed using the baseline values as covariates when necessary, such as when differences in baseline values were found between groups. The screening data for enrolled subjects and those who were not included in this study were compared using Student’s *t*-test. The baseline characteristics of the placebo and APE groups were compared using either Student’s *t*-test or Fisher’s exact test. The values for each test and evaluation item were presented as the mean ± standard deviation or the mean ± standard error. The significance level for the tests was set at 5% (two-sided). Statistical analyses were performed using IBM SPSS Statistics 29 software (International Business Machines Corporation, Armonk, NY, USA) and JMP^®^ 17 software (SAS Institute Inc., Cary, NC, USA).

## 3. Results

### 3.1. Characteristics of the Study Subjects

Screening tests were performed on 635 potential candidates for whom consent was obtained, and 110 subjects were selected. All 110 selected subjects were randomly assigned to the placebo group (N = 55; male, 28; female, 27) or the test food group (N = 55; male, 27; female, 28). A comparison of the screening test endpoints for the enrolled and non-enrolled participants is shown in Table 1. While there was no significant difference in age between the groups, the enrolled participants had a significantly higher proportion of high-SA-βGal CD8+ and CD4+ T cells compared to the non-enrolled group. This suggested that the enrolled subjects represented a cohort with immunosenescence within the 40–50-year-old age range. Additionally, the number of naïve T cells, which typically decrease with age, was significantly lower in the enrolled participants, whereas the numbers of effector memory and TEMRA cells were significantly higher. Furthermore, the proportion of PD-1-positive cells, a marker of exhausted T cells, was significantly higher in the enrolled subjects than in the non-enrolled group. These findings indicated that the enrolled subjects had more advanced immunosenescence relative to their age based on their T cell subset populations.

**Figure 1:**
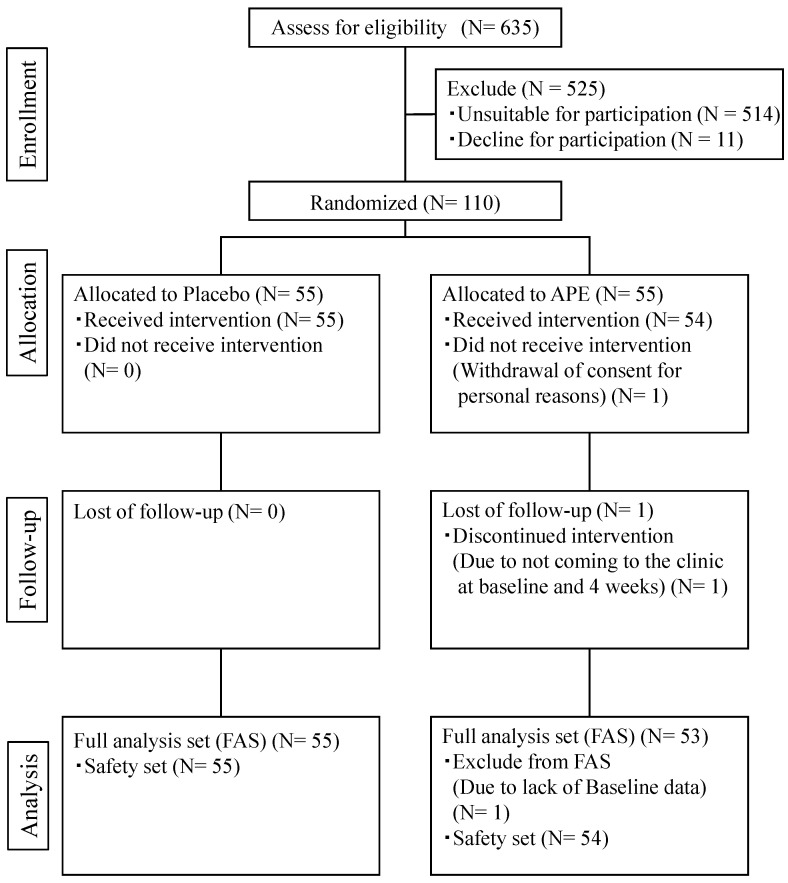
Participant flow.

Of the 110 enrolled subjects, one subject in the APE group withdrew consent to participate in the study before attending the baseline examination and another subject in the food group began taking the test supplement but did not attend the baseline or 4-week examination. The principal investigational doctor determined that it would be difficult for the participants to continue in this study and subsequently terminated their participation. Finally, a total of 108 subjects completed the prescribed study schedule: 55 in the placebo group and 53 in the APE group (Figure 1).

Of the 108 patients who completed the study schedule, one (placebo group) had an 89.3% intake rate of the trial supplement, one (placebo group) had a 96.4% intake rate, four (two in the placebo group and two in the APE group) had a 98.2% intake rate, and the remaining 102 had a 100% intake rate. The average intake rates were 99.7% for the placebo group and 99.97% for the APE group. Of the 110 patients allocated to the trial supplement, 108 with data on primary and secondary endpoints after baseline testing were included in the full analysis set (FAS), representing the largest analysis population. The safety set consisted of 109 patients (55 in the placebo group and 54 in the test food group), excluding one patient in the test food group who did not start taking the trial supplement.

As baseline characteristics for the FAS, Table 2 shows the results for age, height, weight, body mass index, systolic blood pressure, diastolic blood pressure, pulse rate, proportion of high = SA-βGal CD8+ and CD4+ T cells, T cell subsets, proportion of PD-1-expressing cells, and proportions of CD3+, CD4+, and CD8+ lymphocytes at the time of the baseline examination. No significant differences were observed between the placebo and APE groups in these parameters.

### 3.2. Primary and Secondary Outcome Measures

**Figure 2:**
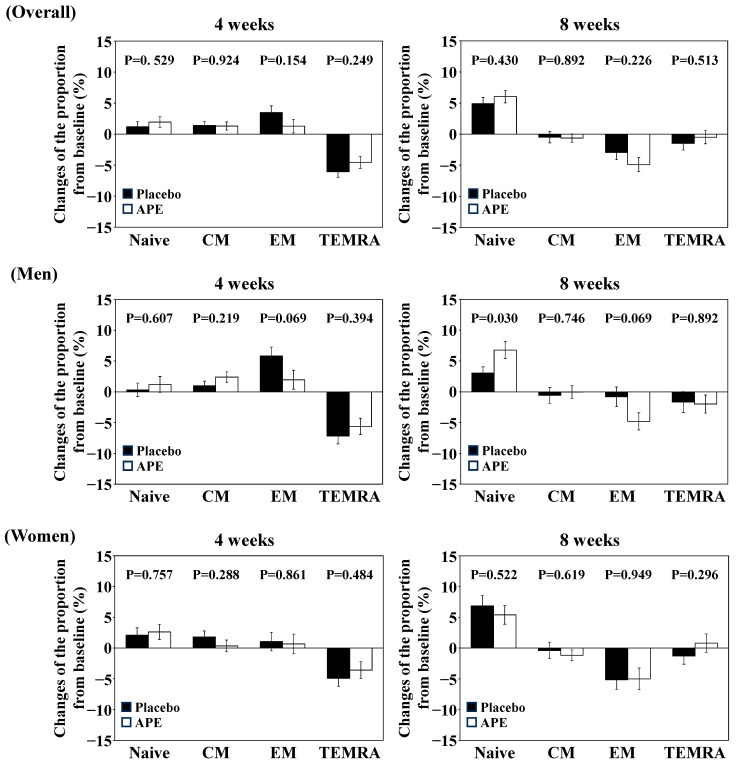
The changes in the proportion of subsets of CD8+ T cells. Values are represented as means ± SDs. Placebo and APE mean Placebo and APE groups, respectively. Note that “4 weeks” and “8 weeks” indicate the time points (in weeks) after the start of trial supplement intake. “Naive”, “CM”, “EM”, and “TEMRA” mean naive T cell, central memory T cell, effector memory T cell, and TEMRA cell, respectively. Student’s *t*-test was used to compare Placebo and APE. The *p*-values are shown in the graphs.

**Figure 3:**
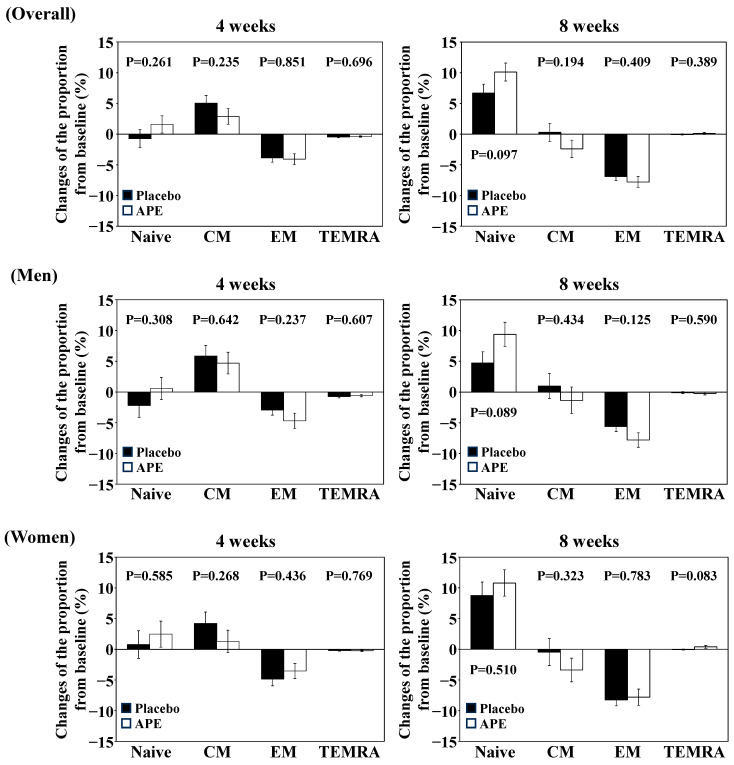
The changes in the proportion of subsets of CD4+ T cells. Values are represented as means ± SDs. Placebo and APE mean Placebo and APE groups, respectively. Note that “4 weeks” and “8 weeks” indicate the time points (in weeks) after the start of trial supplement intake. “Naive”, “CM”, “EM”, and “TEMRA” means naive T cell, central memory T cell, effector memory T cell, and TEMRA cell, respectively. Student’s *t*-test was used to compare Placebo and APE. The *p*-values are shown in the graphs.

The changes in the values of the primary and secondary endpoints from baseline are shown in Table 3 and Table S1 and Figure 2 and Figure 3. The proportion of high-SA-βGal CD8+ T cells decreased after treatment intake in both the placebo and APE groups, with no significant differences between groups (Table 3). As for the secondary endpoints, the proportion of naïve CD4+ T cells tended to increase in the APE group and the proportion of CD3+ T cells in lymphocytes tended to be higher in the placebo group than in the APE group; however, no significant differences were observed (Table S1, Figure 2). For the proportion of PD-1-positive CD8+ T cells, both groups showed approximately 6% higher proportions at eight weeks compared to the baseline. The evaluation of PD-1-positive CD8+ T cell measurements at the 8-week point was put on hold due to the lack of consistency in the data compared to other time points (Table S1).

### 3.3. Subgroup Analysis

The results of the subgroup analysis according to sex are shown in Table 3 and Table S1, Figure 2 and Figure 3. In terms of the proportion of high-SA-βGal CD8+ T cells in male subjects, both groups showed a decrease at 8 weeks from the baseline; however, the APE group showed significantly lower values than the placebo group at the same period (Table 3). In contrast, for females, both groups showed a decrease at 8 weeks from the baseline, but there was no significant difference between the groups (Table 3).

The CD8+ T cell/lymphocyte ratio was significantly lower in males in the placebo group than in the APE group at baseline, resulting in significantly higher baseline values for the CD4+/CD8+ cell ratio in the placebo group than in the APE group (Table S1B). Therefore, an ANCOVA with the baseline value as a covariate was conducted. No differences were observed between the placebo and APE groups in the CD8+ T cell/lymphocyte ratio and the CD4+/CD8+ ratio (*p* = 0.6546 and 0.1378, respectively). Although a significant difference was observed in the CD4+/CD8+ ratio among females (*p* = 0.048), the ANCOVA showed no significant difference (*p* = 0.0875).

For the T cell subset, the proportion of naïve CD8+ T cells in males increased in both groups after the start of intake but became significantly higher in the APE group than in the placebo group at 8 weeks (Table S1, Figure 2). In contrast, the proportion of effector memory CD8+ T cells decreased from baseline to 8 weeks in the APE group, whereas the placebo group showed little change from baseline. At 8 weeks, the APE group showed a lower value than the placebo group (ANCOVA, *p* = 0.0462). This suggests that the increase in the number of naïve T cells was due to the removal of senescent effector memory CD8+ T cells. No significant differences between the groups were observed in other parameters or in females (Table S1, Figure 2).

### 3.4. Exploratory Efficacy Analysis

For exploratory efficacy evaluation, the gene expression levels of p16, p21, p53, and Sirt1 in the PBMC population were measured using absolute quantification via dPCR. The results showed no movement linked to changes in high-SA-βGal CD8+ T cells (Table S2).

### 3.5. Safety

Table 4 lists the adverse events observed in this study. There were 15 adverse events reported in 14 of 55 subjects in the placebo group and 22 adverse events in 14 of the 54 subjects in the APE group.

All adverse events were mild, and the symptoms were resolved during this study. The principal investigational doctor judged that these adverse events were unrelated to the trial supplements and that no side effects were observed. Additionally, although significant changes were observed in the individual clinical test values and the average clinical test values for each group, the principal investigational doctor judged these changes to be clinically insignificant.

## 4. Discussion

Geroscience is a concept that revolves around the notion that targeting the aging process can have the greatest impact on human health because aging is the greatest risk factor for many diseases [37]. Cellular senescence, one of the 15 hallmarks of aging, is vital because of its impact on aging through the cessation of cell proliferation, decline in cell function, and secretion of SASP factors [11]. Senolytic agents have been studied as a means of combating cellular senescence. For example, quercetin plus dasatinib, BCL2 inhibitors, BET (bromodomain and extra-terminal domain) inhibitors, and fisetin are expected to act as senolytics [14,38,39]. Although studies have investigated senolytic agents and their anti-aging effects in humans, their number remains limited [40]. *A. pilosa* Ledeb., a traditional medicinal herb, has been confirmed to have senolytic effects in vivo, making it a promising candidate as an oral senolytic food ingredient [32]. However, like other potential senolytic agents, its effects on humans remain unclear. Therefore, we conducted a randomized, double-blind, placebo-controlled, parallel-group comparison study in which middle-aged Japanese subjects with immunosenescence continuously consumed APE for eight weeks.

The efficacy of APE was not observed in the overall population; however, APE intake was suggested to reduce high-SA-βGal CD8+ T cells in the male population. The populations of high-SA-βGal CD8+ T cells were presumed to be memory T cells and TEMRA T cells. Additionally, among memory T cells, effector memory T cells express higher levels of SA-βGal than central memory T cells [23]. In this study, the consumption of APE resulted in little change in the proportion of central memory T cells; however, the proportion of effector memory T cells decreased while that of naïve T cells increased. This suggests that the consumption of APE may eliminate effector memory CD8+ T cells that highly express SA-βGal and are undergoing aging. Martínez-Zamudio et al. reported that the high-SA-βGal CD8+ T cell population showed characteristics of senescence induced by telomere dysfunction and p16-mediated senescence. Based on this, the reduction in high-SA-βGal CD8+ T cells observed with APE intake in this study was presumed to reflect its senolytic effects. However, to more accurately evaluate the senolytic effects of APE, it would be appropriate to assess senescent T cell markers (e.g., KLRG1 (killer cell lectin-like receptor G1)), and SASP factors would be useful for evaluating the senolytic effects of APE [41].

In a previous study, the administration of APE to aged mice was reported to decrease the gene expression levels of p16 and p53 [32]. However, in this study, we observed no impact of APE intake on the expression of these genes and Sirt1. One possible explanation is that APE exerts only a limited effect on senescent cells. Another consideration is that the PBMC population used here for gene expression analysis was composed not only of CD3+ T cells but also B cells, NK cells, monocytes, and dendritic cells. The presence of these additional cell types, beyond CD8+ T cells, may have obscured any potential APE-induced effects [42].

In this study, a decrease in high-SA-βGal CD8+ T cells, an increase in naïve CD8+ T cells, and a decrease in effector memory CD8+ T cells were observed in males following APE intake, whereas these effects were not observed in females. In women, hormones such as estrogen (specifically, estradiol), progesterone, and their regulatory factors (e.g., follicle-stimulating hormone [FSH] and luteinizing hormone [LH]) have well-documented effects on immune cell function and senescence [43,44,45,46]. Estrogen, for instance, not only modulates the expression of senescence-related proteins but also influences the differentiation and activation of T cells [44,45]. Estrogen receptors are also expressed on CD8+ T cells, and it has been reported that estrogen analogs can induce antitumor immunity [47]. Indeed, the fluctuation in serum levels of these hormones between premenopausal, perimenopausal, and postmenopausal women could result in variable responses to senolytic interventions with APE. For instance, Fang et al. reported that when fisetin and dasatinib plus quercetin were administered to male and female mice, respectively, the treatment was effective in reducing SASP in male mice, whereas it had no effect or even a harmful effect in female mice [48]. They speculated that the difference in the effectiveness of these senolytics between males and females may be due to the slower biological aging process in females compared to males [48]. Since sex hormones, particularly estrogen, have been shown to have anti-aging effects, future research should measure sex hormone levels, such as estrogen, and investigate their potential influence on the effects of APE on senescent cells.

The targets of senolytic agents have been proposed to include anti-apoptotic proteins of senescent cells (e.g., the BCL-2 family), organelles such as mitochondria that have become dysfunctional in senescent cells, surface antigens of senescent cells, membrane potentials, and other specific biochemical changes [49,50]. Dasatinib, a tyrosine kinase inhibitor and a specific inhibitor of the anti-apoptotic proteins BCL-2 and BCL-xL, induces apoptosis by suppressing the ERK-AKT signaling pathway, thereby inducing senolysis [51,52]. *A. pilosa* Ledeb. contains over 250 substances, including flavonoids, phenolic compounds, phloroglucinol derivatives, tannins, and pentacyclic triterpenoids [24,25]. It has been reported that APE and its constituent compounds induce apoptosis in cancer cells by activating Bax and caspase-3 and inhibiting the ERK-AKT signaling pathway [53,54]. Dryocrassin ABBA, a phloroglucinol derived from *Dryopteris crassirhizoma*, induces apoptosis in liver cancer cell lines by simultaneously inhibiting BCL-2 expression and increasing Bax expression [55]. Agrimols, a characteristic phloroglucinol contained in APE, also reduce the proportion of SA-βGal-positive doxorubicin-induced senescent WI-38 cells by inducing apoptosis. Therefore, we speculated that agrimols are the active substances responsible for the senolytic effects of APE [32]. The mechanism of APE senolysis is not clear, but APE may exhibit senolysis via a mechanism similar to that of dasatinib and navitoclax (ABT-263).

Mitochondrial function is impaired in senescent cells, so it can be targeted by senolytic treatments. Agrimol B activates the Sirtuin/Nrf2 pathway [56]. Nrf2 activation contributes to mitochondrial biogenesis and energy production, enhancing mitochondrial function [57,58]. Therefore, agrimol B may indirectly enhance mitochondrial function. In contrast, agrimol B has been reported to induce apoptosis in tumor cells by inhibiting mitochondrial function by binding to PGC-1α (peroxisome proliferator-activated receptor gamma coactivator 1-alpha) [59]. Thus, although agrimol B may have opposing effects on mitochondria, it may induce apoptosis in senescent cells by inhibiting mitochondrial function.

Although various studies on senolytic agents have been conducted, few reports have verified their effects in humans [6]. In this context, the results of this study suggesting the senolytic effects of APE intake are expected to serve as preliminary insights for the development of senolytic agents. However, further investigation is needed to clarify the senolytic effects of APE. Moreover, even if the senolytic effects of APE are confirmed, its impact on age-related diseases remains unclear. For example, patients with bipolar disorder show decreased proportions of naïve T cells, increased proportions of memory and senescent T cells, and inflammation throughout the body [60]. Additionally, cytotoxic CD8+ cells can infiltrate the brain and have been implicated in inflammatory and degenerative brain diseases [61]. These reports indicate that the clearance of senescent CD8+ T cells by APE may affect the central nervous system. Indeed, our research group has confirmed that the administration of APE improves mood states such as vigor and fatigue in middle-aged Japanese people experiencing poor mood states [62]. Given these preliminary findings, further investigation into the senolytic effects of APE, particularly in the context of sex-specific hormonal influences, is warranted.

## 5. Conclusions

In this study, an *A. pilosa* extract containing agrimols (APE) was administered to men and women aged 40 to under 60 years with immunosenescence. Our findings indicated that the intake of APE in the overall population did not significantly reduce senescent immune cells (high-SA-βGal CD8+ T cells). However, a potential reduction in high-SA-βGal CD8+ T cells was observed in men, suggesting a possible sex-dependent effect of APE on immunosenescence. The effect of APE on the expression of cell cycle markers and the *Sirt1* gene was not observed, which was likely due to the heterogeneous cell population used for measurement. These results, while preliminary, underscore the importance of evaluating additional senescence markers, SASP factors, and oxidative stress markers to clarify the mechanism of APE’s effects. Moreover, since the degree and mechanism by which APE reduces senescent cells may differ between men and women, further investigation is needed into potential sex differences, including the measurement of sex hormones such as estrogen and progesterone, in future studies.

Furthermore, our finding highlights the need to integrate mechanistic in vitro studies, in vivo animal experiments, and larger-scale human trials to determine whether APE can effectively mitigate immunosenescence and serve as a targeted intervention for age-related immune decline.

## 6. Limitations

This study has several limitations. First, the effectiveness of APE (containing agrimols) in individuals aged 60 and above remains unclear, as the current investigation focused on those aged 40 to under 60. Second, although a reduction in high-SA-βGal CD8+ T cells was found only in males, the factors contributing to this potential sex-specific response—particularly in females—were not fully explored. Third, we focused on SA-βGal as a senescent marker without assessing other markers such as KLRG1, oxidative stress markers, and SASP factors (e.g., IL-1β, IL-6), making it difficult to obtain a comprehensive picture of APE’s effect on the senescence phenotype. Similarly, the effects of APE on cell cycle markers in the CD8+ T cell population could not be investigated, leaving the mechanisms underlying its potential senolytic activity unresolved. Fourth, while this study highlights an impact on cellular senescence within the context of immunosenescence, its relevance to aging-related diseases remains unknown. Thus, additional research is needed to clarify whether APE can modulate aging pathways beyond immunosenescence parameters.

Furthermore, this study did not systematically evaluate the role of sex hormones such as estrogen and progesterone, which may affect the senolytic effects. Accounting for these hormonal influences is critical to fully understanding the observed sex-specific tendency of APE’s effects. Future studies should include a more diverse age range, multiple senescence markers and SASP factors, and detailed investigations of mitochondrial function and apoptosis-related molecules (e.g., BCL-2, Bax). Such comprehensive studies will help elucidate the mechanisms underlying APE’s potential senolytic effects, determine whether distinct effects exist between males and females, and establish its potential utility in mitigating both immunosenescence and age-related disease. Therefore, further research with improvements in study design and evaluation parameters is necessary to validate and expand upon our preliminary findings.

## Figures and Tables

**Table 1:** Comparison of the population of T cells and their subsets and immunosenescence markers between enrolled and non-enrolled subjects.

		Overall		Non-Enrolled		Enrolled		
		N = 632		N = 522		N = 110		
Items		Mean	SD	Mean	SD	Mean	SD	*p* Value
Age (years)		50.2	5.2	50.2	5.3	50.0	4.9	0.6057
CD8+	High SA-βGal (%)	65.46	20.07	61.05	19.26	86.36	5.08	<0.0001
	Naïve (%)	34.56	17.50	38.29	16.79	16.86	6.56	<0.0001
	Central memory (%)	21.51	9.37	21.91	9.29	19.59	9.57	0.0182
	Effector memory (%)	30.09	12.52	27.93	11.64	40.31	11.48	<0.0001
	TEMRA (%)	13.85	11.69	11.87	10.12	23.24	13.93	<0.0001
	PD-1+ (%)	9.815	5.834	9.710	5.640	10.312	6.685	0.3255
CD4+	High SA-βGal (%)	52.32	17.78	49.94	17.36	63.62	15.27	<0.0001
	Naïve (%)	19.94	10.07	20.97	10.07	15.04	8.52	<0.0001
	Central memory (%)	62.93	9.56	62.79	9.58	63.63	9.49	0.3987
	Effector memory (%)	16.78	7.74	15.95	7.41	20.73	8.09	<0.0001
	TEMRA (%)	0.348	0.800	0.297	0.673	0.593	1.210	0.0004
	PD-1+ (%)	12.407	4.948	12.124	4.995	13.747	4.507	0.0017
CD3+/Lymphocyte (%)		78.01	8.41	77.86	8.53	78.71	7.81	0.3379
CD4+/Lymphocyte (%)		52.94	8.74	53.45	8.70	50.47	8.57	0.0011
CD8+/Lymphocyte (%)		19.12	6.59	18.68	6.37	21.23	7.19	0.0002
CD4+/CD8+		3.20	1.51	3.30	1.53	2.76	1.30	0.0007

Values are represented as means ± SDs. “Overall” is the total subject population for which consent was obtained, “Non-enrolled” is the population that was deemed ineligible for participation or withdrew consent during screening or testing, and “Enrolled” is the population of subjects who were enrolled. Student’s *t*-test was used to compare Enrolled and Non-enrolled.

**Table 2:** Demographic and baseline characteristics of full analysis set.

			Placebo		APE		
			N = 55		N = 53		
			Mean	SD	Mean	SD	*p* Value
Sex	Male	subjects	28		25		0.705
	Female	subjects	27		28		
Age		years	50.1	4.8	50	4.9	0.970
Height		cm	164.47	8.27	164.45	6.72	0.987
Body weight		kg	59.65	11.75	57.32	8.24	0.238
BMI		kg/m^2^	21.88	2.86	21.14	2.34	0.145
Systolic blood pressure		mmHg	114.5	13.3	112.7	11.1	0.430
Diastolic blood pressure		mmHg	72.1	11.3	70.5	9.9	0.452
Pulse rate		bpm	69.9	8.2	68.4	9.5	0.372
Proportion to Lymphocyte	CD3+ T cells	%	75.14	8.37	77.27	6.95	0.155
	CD4+ T cells	%	46.74	8.54	46.63	9.43	0.946
	CD8+ T cells	%	21.85	8.06	23.46	9.02	0.328
CD4+/CD8+			2.533	1.241	2.423	1.337	0.659
CD8+ T cells	High SA-βGal	%	84.84	7.66	85.16	8.85	0.840
	Naïve	%	18.47	9.43	17.54	7.65	0.578
	Central memory	%	16.21	10.29	14.07	7.6	0.221
	Effector memory	%	38.89	11.77	39.8	12.18	0.694
	TEMRA	%	26.43	14.9	28.59	13.91	0.439
	PD-1+	%	8.180	6.040	7.680	5.050	0.638
CD4+ T cells	High SA-βGal	%	53.7	17.59	54.83	16.47	0.732
	Naïve	%	24.36	12.71	22.8	10.44	0.489
	Central memory	%	53.57	11.61	52.81	10.95	0.727
	Effector memory	%	21.09	7.77	23.32	10.61	0.218
	TEMRA	%	0.981	2.062	1.071	1.549	0.798
	PD-1+	%	14.360	4.820	14.980	5.580	0.542

Values are represented as means ± SDs. Placebo and APE mean Placebo and APE groups, respectively. Student’s *t*-test was used to compare Placebo and APE. Sex was compared using Fisher’s exact test.

**Table 3:** The changes in the proportion of high-SA-βGal-expressing T cells.

					Time After Start of Intake of Trial Supplement					
					Baseline		4 Weeks		8 Weeks	
					Mean	SE	Mean	SE	Mean	SE
Overall	High-SA-βGal CD8+	%	Placebo	N = 55	84.84	1.03	−2.07	1.01	−5.25	1.20
			APE	N = 53	85.16	1.22	−3.02	0.96	−6.92	1.12
			*p* value		0.840		0.499		0.311	
	High-SA-βGal CD4+	%	Placebo	N = 55	53.70	17.59	−6.37	1.84	−15.82	1.41
			APE	N = 53	54.83	16.47	−7.29	1.70	−16.91	1.67
			*p* value		0.732		0.715		0.618	
Male	High-SA-βGal CD8+	%	Placebo	N = 28	85.06	1.26	−1.13	1.26	−4.01	1.18
			APE	N = 25	86.08	1.31	−3.00	1.27	−8.20	1.69
			*p* value		0.575		0.303		0.044	
	High-SA-βGal CD4+	%	Placebo	N = 28	53.09	3.62	−4.82	2.07	−14.19	1.78
			APE	N = 25	55.00	2.94	−7.11	2.15	−16.37	1.88
			*p* value		0.689		0.447		0.404	
Female	High-SA-βGal CD8+	%	Placebo	N = 27	84.61	1.67	−3.05	1.60	−6.54	2.12
			APE	N = 28	84.34	2.00	−3.04	1.44	−5.78	1.47
			*p* value		0.918		0.997		0.770	
	High-SA-βGal CD4+	%	Placebo	N = 27	54.34	3.10	−7.97	3.09	−17.50	2.20
			APE	N = 28	54.68	3.44	−7.45	2.62	−17.39	2.71
			*p* value		0.941		0.897		0.975	

Values are represented as means ± SEs. Placebo and APE mean Placebo and APE groups, respectively. Between-group comparisons were assessed by Student’s *t*-tests. The baseline values represent the measured values, while the values at 4 and 8 weeks indicate changes from baseline.

**Table 4:** List of adverse events.

		Placebo	APE
		N = 55	N = 54
Proportion of subjects with adverse events	%	25.5	25.9
Total number of adverse events	cases	15 (3)	22 (3)
COVID-19	cases	0	1 (1)
Influenza A (headache)	cases	0	1
Meibomian gland infarction (lower left eyelid)	cases	0	1 (1)
Common cold symptoms (sore throat)	cases	0	1
Cold-like symptoms (fatigue)	cases	0	1 (1)
Cold-like symptoms (fatigue, nasal discharge)	cases	0	1
Common cold symptoms (fatigue, fever)	cases	1	0
Common cold symptoms (sore throat, cough, joint pain)	cases	1	0
Sore throat, nasal discharge	cases	0	1
Nasal discharge	cases	2	1
Nasal discharge, headache	cases	0	1
Cough	cases	1 (1)	0
Cough, hoarseness	cases	0	1
Fever	cases	1	1
Fever, cough	cases	1	0
Gingivitis	cases	1	0
Stomach pain	cases	1	0
Abdominal pain	cases	0	2
Soft stool	cases	1	0
Diarrhea	cases	2	0
Occipital neuralgia	cases	0	1
Headache	cases	2 (2)	3
Headache, vomiting	cases	1	0
Migraine	cases	0	1
Dizziness	cases	0	1
Vertigo	cases	0	1
Fatigue	cases	0	1
Joint pain (shoulder, arm)	cases	0	1

Numbers in parentheses indicate adverse events that occurred prior to intake of trial supplements.

## Data Availability

The data presented in this study are available on request from the corresponding author due to privacy and ethical reasons.

## Supplementary Materials

The following supporting information can be downloaded at: https://www.mdpi.com/article/10.3390/nu17040667/s1, Table S1: The Changes in T lymphocyte proportions and its subsets; Table S2: The gene expression of cell cycle markers and Sirt1 in the PBMC population.
